# Phototunable Magnetism in Copper Octacyanomolybdate

**DOI:** 10.1155/2014/762765

**Published:** 2014-05-08

**Authors:** Jun Ohara, Shoji Yamamoto

**Affiliations:** Department of Physics, Hokkaido University, Sapporo 060-0810, Japan

## Abstract

We introduce copper molybdenum cyanides of general formula Cu_2_[Mo(CN)_8_]*·n*H_2_O, which can serve as optofunctional magnetic devices. Their ground states generally stay paramagnetic down to temperatures of the K order but exhibit a spontaneous magnetization upon photoirradiation usually below a few tens of K. To interest us still further, such a ferromagnetic stateinduced by blue-laser irradiation is demagnetized step by step through further application of red or near-infrared laser pulses. We solve this intriguing photomagnetism. The ground-state properties are fully revealed by means of a group-theoretical technique. Taking account of experimental observations, we simulate applying pump laser pulses to a likely ground state and successfully reproduce both the magnetization and demagnetization dynamics. We monitor the photorelaxation process through angle-resolved photoemission spectroscopy. Electrons are fully itinerant in any of the photoinduced steady states, forming a striking contrast to the initial equilibrium state of atomic aspect. The fully demagnetized final steady state looks completely different from the initial paramagnetism but bears good analogy to one of the possible ground states available with the Coulomb repulsion on Cu sites suppressed.

## 1. Introduction

Photomanipulation of magnetism in metal-based molecular systems is one of the most challenging themes in the fields of synthetic chemistry, solid-state physics, and engineering. Many efforts to developments of new materials and measurement techniques have been devoted to an achievement of the purpose. Prussian-blue analogues which involve hexacyanometalates [M(CN)_6_]^*n*−^ (*M*: 3*d* transition metals) are widely known as phototunable magnets [1–4]. In the iron-cobalt analogues, photoirradiation triggers the transition from the low-spin configuration, Fe^II^(*S* = 0)–CN–Co^III^(*S* = 0), to the high-spin configuration, Fe^III^(*S* = 1/2)–CN–Co^II^ (*S* = 3/2) [5–7]. Such a photoredox reaction, which is induced by the charge-transfer excitation between cyano-bridged metals, is one of the key ingredients of photomagnetism in metal-based molecular systems [8]. For the purpose of designing and constructing new photoswitchable magnets based on the octacyanometalates [*M*(CN)_8_]^*n*−^ (*M* = Mo, W), the chemical explorations of various cationic precursor complexes have made remarkable progress in the past decade [9, 10].

Octa-coordinated complexes are rich in configuration and such a geometric variety is characteristic of transition metals of the second and third rows [11]. The octacyano-bridged systems possess various charge-transfer and magnetic-exchange pathways and the resultant conducting and magnetic properties. Paramagnetic [12, 13], ferromagnetic [14–16], and antiferromagnetic [17–19] assemblies have been actually synthesized. Among others, octacyanomolybdates of [Mo^IV^(CN)_8_]^4−^-based coordination networks are much interesting, where the anionic precursor as a building block takes the square antiprism (SAPR-8) configuration [20, 21].

**Figure 1:**
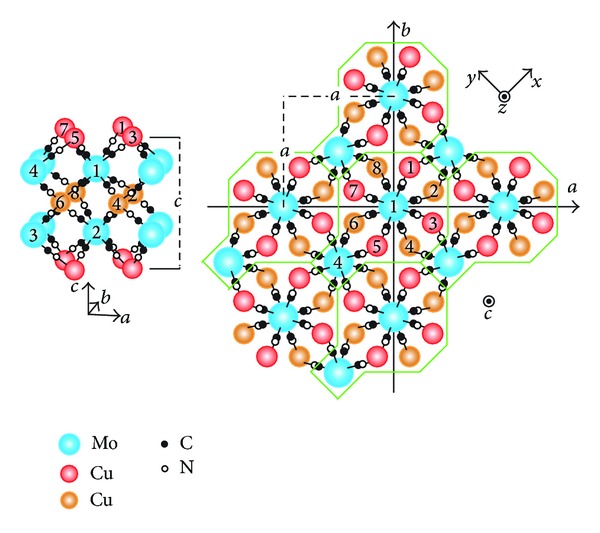
The cyano-bridged copper-molybdenum bimetallic assembly Cu_2_[Mo(CN)_8_]. Cu and Mo sites in each cell are labeled in this manner.

Recently, remarkable phototunable magnetism of the copper-molybdenum bimetallic assemblies Cu_2_[Mo(CN)_8_] · *n*H_2_O [22–25] has been reported. Ohkoshi et al. [26] observed details of the photoinduced magnetization and demagnetization of Cu_2_[Mo(CN)_8_] · 8H_2_O. At the sufficiently low temperature 3 K, irradiation of a paramagnetic ground state with a laser light of 473 nm induces a macroscopic magnetization. Subsequent irradiation with another laser light of 658 nm conversely reduces the produced magnetization. When the sample is further irradiated with 785 nm and 840 nm laser lights successively, the magnetization gets smaller and smaller. The photoinduced magnetization is stable for hours up to 100 K or more and the photoconversion, both magnetization and demagnetization, is repeatable. In spite of such intriguing observations, neither the photoexcitation mechanism nor the ground-state magnetism has been interpreted yet beyond a phenomenological understanding. As is shown in Figure 1, the mixed-valent bimetallic compound Cu_2_^II^[Mo^IV^(CN)_8_] crystallizes in the *I*4/*m* tetragonal space group with **C**_4*h*_ point, where each constituent ion [Mo^IV^(CN)_8_]^4−^ takes the SAPR-8 configuration with **D**_4*d*_ point symmetry. Fully filled 4*d*_*z*^2^_ orbitals on Mo^IV^ sites and half-filled 3*d*_*yz*/*zx*_ orbitals on Cu^II^ sites are physically active due to the crystalline-field splitting. The supramolecular coordination network based on octacyanomolybdates of the SAPR-8 configuration gives unique hopping paths and they must play a major role in realizing the photoreversible ferromagnetism.

We reveal possible magnetic and nonmagnetic states by making good use of a group-theoretical analysis on the *I*4/*m* cyano-bridged bimetallic assembly and simulate photoirradiation of the equilibrium states by numerically solving the time-dependent Schrödinger equation.

## 2. Model Hamiltonian and Its Symmetry Properties

We describe the supramolecular coordination network comprising divalent copper and tetravalent molybdenum ions in terms of a three-band extended Hubbard Hamiltonian [27, 28] of 2/3 electron filling:

(1) H=∑n,σ[∑i=18(εCunn:Cu(i)σ+UCu2nn:Cu(i)σnn:Cu(i)−σ)+∑j=14(εMonn:Mo(j)σ+UMo2nn:Mo(j)σnn:Mo(j)−σ)] +∑〈n,m,i,j〉 ‍∑σ,τ{JMoCucn:Cu(i)σ†cm:Mo(j)τ†cn:Cu(i)τcm:Mo(j)σ‍+JMoCu′2(cn:Cu(i)σ†cn:Cu(i)τ†cm:Mo(j)τ×cm:Mo(j)σ+H.c.)+tMoCu2[(−1)i+j+1cn:Cu(i)σ†cm:Mo(j)σ+H.c.]+VMoCunn:Cu(i)σnm:Mo(j)τ} +∑〈n≠m〉 ∑i=14 ∑σ,τ[JCuCucn:Cu(i)σ†cm:Cu(i+4)τ†cn:Cu(i)τcm:Cu(i+4)σ‍+JCuCu′2(cn:Cu(i)σ†cn:Cu(i)τ†cm:Cu(i+4)τ×cm:Cu(i+4)σ+H.c.)+tCuCu2(cn:Cu(i)σ†cm:Cu(i+4)σ+H.c.)+VCuCunn:Cu(i)σnm:Cu(i+4)τ],

with *n*_**n**:*A*(*i*)_ = ∑_*σ*_ *n*_**n**:*A*(*i*)*σ*_ = ∑_*σ*_ *c*_**n**:*A*(*i*)*σ*_^†^ *c*_**n**:*A*(*i*)*σ*_, where *c*_**n**:*A*(*i*)*σ*_^†^ creates an electron of spin *σ* = ↑, ↓≡± in the Cu 3*d*_*zx*_ (*A* = Cu; *i* = 1,2, 5,6), Cu 3*d*_*yz*_ (*A* = Cu; *i* = 3,4, 7,8), or Mo 4*d*_*z*^2^_ (*A* = Mo; *i* = 1,2, 3,4) orbitals labelled *i* at unit cell **n**, as is shown in Figure 1. An on-site Coulomb repulsion and an orbital energy on the Cu(Mo) site are described as *U*_Cu(Mo)_ and *ε*_Cu(Mo)_, respectively. ∑_〈**n**,**m**,*i*,*j*〉_ and ∑_〈**n**≠**m**〉_∑_*i*=1_^4^ mean the sums all over nearest neighboring copper and molybdenum sites and nearest neighboring copper sites, respectively. The electron hoppings *t*_MoCu/CuCu_, the intermetallic Coulomb repulsions *V*_MoCu/CuCu_, the exchange interactions *J*_MoCu/CuCu_, and the pair-hopping interactions *J*_MoCu/CuCu_′ are considered. All the two-body terms are treated within the Hartree-Fock (HF) scheme.

**Figure 2:**
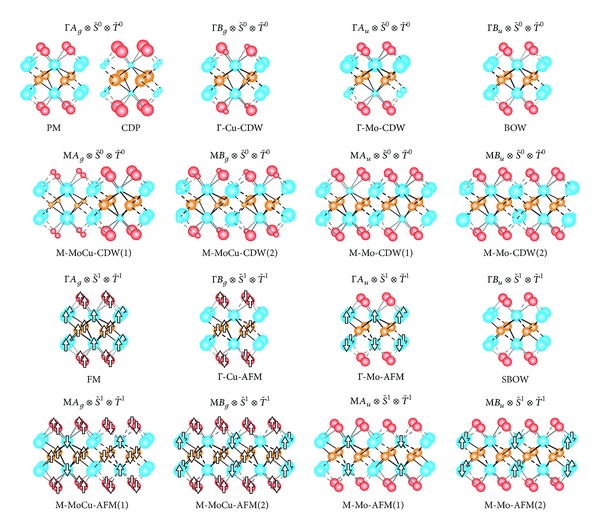
Possible nonmagnetic [$\Gamma \overset{˘}{D}(\Gamma )⊗{\overset{˘}{S}}^{0}⊗{\overset{˘}{T}}^{0}$ and $\text{M}\overset{˘}{D}(\text{M})⊗{\overset{˘}{S}}^{0}⊗{\overset{˘}{T}}^{0}$] and magnetic [$\Gamma \overset{˘}{D}(\Gamma )⊗{\overset{˘}{S}}^{1}⊗{\overset{˘}{T}}^{1}$ and $\text{M}\overset{˘}{D}(\text{M})⊗{\overset{˘}{S}}^{1}⊗{\overset{˘}{T}}^{1}$] states, where varied circles and arrows represent oscillating electron and spin densities, respectively, and varied segments in the nonmagnetic and magnetic states describe oscillating bond orders and spin bond orders, respectively. They consist of paramagnetism (PM) and charge disproportionation (CDP) of full symmetry, charge density waves (CDWs) within the molybdenum and/or copper sublattices, a bond order wave (BOW), ferromagnetism (FM), antiferromagnetism (AFM) within the molybdenum and/or copper sublattices, and a spin bond order wave (SBOW).

Unless the gauge symmetry is broken, the symmetry group of any electron system can be written as [29, 30]

(2) $$\begin{array}{c} G=P\times S\times T, \end{array}$$

where **P**, **S**, and **T** are the groups of space, spin rotation, and time reversal, respectively. The space group is further decomposed as

(3) $$\begin{array}{c} P=L\wedge D, \end{array}$$

which read {*l*_*a*_ **a** + *l*_*b*_ **b** + *l*_*c*_ **c**; *l*_*a*_, *l*_*b*_, *l*_*c*_ ∈ **Z**} ≡ **L** and {*E*, *C*_4*z*_, *C*_2*z*_, *C*_4*z*_^−1^, *I*, *IC*_4*z*_, *IC*_2*z*_ ≡ *σ*_*h*_, *IC*_4*z*_^−1^} ≡ **C**_4*h*_, respectively. An irreducible representation of **G** over the real number field, which is referred to as $\overset{˘}{G}$, consists of those of **P**, **S**, and **T**: $\overset{˘}{G}=\overset{˘}{P}⊗\overset{˘}{S}⊗\overset{˘}{T}$. Once a wave vector **Q** is fixed, the relevant little group **D**(**Q**) is given. $\overset{˘}{P}$ is therefore labeled as $Q\overset{˘}{D}(Q)$. Since cell doubling along the *a* and *b* directions is expected under the present lattice structure, we consider **Q** = (*π*/*a*, *π*/*a*, 0) as well as **Q** = 0, which are referred to as M and Γ, respectively, whose little groups are **C**_4*h*_ in itself. A spin rotation by *θ* around an axis **e** (|**e** | = 1), *u*(**e**, *θ*) ∈ **S**, is represented as *u*(**e**, *θ*) = *τ* ^0^cos⁡(*θ*/2) − *i*(**τ** · **e**)sin(*θ*/2), where *τ* ^0^ is the 2 × 2 unity matrix and **τ** = (*τ* ^*x*^, *τ* ^*y*^, *τ* ^*z*^) is a vector composed of the Pauli matrices. The relevant representations of **S** are given by ${\overset{˘}{S}}^{0}(u(e,\theta ))=1$ (singlet) and ${\overset{˘}{S}}^{1}(u(e,\theta ))=O(u(e,\theta ))$ (triplet), where *O*(*u*(**e**, *θ*)) is the 3 × 3 orthogonal matrix satisfying *u*(**e**, *θ*)**τ** ^*λ*^ *u* ^†^(**e**, *θ*) = ∑_*λ*′=*x*,*y*,*z*_[*O*(*u*(**e**, *θ*))]_*λλ*′_ **τ** ^*λ*′^  (*λ* = *x*, *y*, *z*). Those of **T** are given by ${\overset{˘}{T}}^{0}(t)=1$ (symmetric) and ${\overset{˘}{T}}^{1}(t)=-1$ (antisymmetric). Static density-wave solutions are derived from $\Gamma \overset{˘}{D}(\Gamma )⊗{\overset{˘}{S}}^{0}⊗{\overset{˘}{T}}^{0}$, $\text{M}\overset{˘}{D}(\text{M})⊗{\overset{˘}{S}}^{0}⊗{\overset{˘}{T}}^{0}$, $\Gamma \overset{˘}{D}(\Gamma )⊗{\overset{˘}{S}}^{1}⊗{\overset{˘}{T}}^{1}$, and $\text{M}\overset{˘}{D}(\text{M})⊗{\overset{˘}{S}}^{1}⊗{\overset{˘}{T}}^{1}$. The space groups of the former two and the latter two turn out to be **L**_1_∧**C**_4*h*_ and **L**_2_∧**C**_4*h*_, respectively, where **L**_1_ = **L** and **L**_2_ = {(*l*_+_ + *l*_−_)**a** + (*l*_+_ − *l*_−_)**b** + *l*_*c*_ **c**; *l*_+_, *l*_−_, *l*_*c*_ ∈ *Z*}. We explain them in Table 1 and Figure 2. Here, the two-dimensional representations *E*_*g*_ and *E*_*u*_, generally available in a **C**_4*h*_ Hamiltonian, are discarded, because they have no* axial* isotropy subgroup [31, 32]. Every irreducible representation is guaranteed to yield a stable solution only when its isotropy subgroup possesses a one-dimensional fixed point subspace [33].

As is shown in Figure 2, all the nonmagnetic phases corresponding to the $\Gamma \overset{˘}{D}(\Gamma )⊗{\overset{˘}{S}}^{0}⊗{\overset{˘}{T}}^{0}$ and $\text{M}\overset{˘}{D}(\text{M})⊗{\overset{˘}{S}}^{0}⊗{\overset{˘}{T}}^{0}$ representations are classified into paramagnetic (PM) and charge-disproportionate (CDP) states of full symmetry, Γ-type (uniform) and M-type (cell-doubled) charge-density-wave (CDW) states, and a bond-order-wave (BOW = bond-centered CDW) state. There exists a variety of charge-ordering patterns: a CDW on the Cu sublattice (Γ-Cu-CDW), CDWs on the Mo sublattice [Γ-Mo-CDW, M-Mo-CDW(1), and M-Mo-CDW(2)], and CDWs on both the Mo and Cu sublattices [M-MoCu-CDW(1) and M-MoCu-CDW(2)]. They will be stabilized by predominant electron-phonon couplings or relevant intermetallic Coulomb interactions [34, 35]. We further find varied magnetic phases corresponding to the $\Gamma \overset{˘}{D}(\Gamma )⊗{\overset{˘}{S}}^{1}⊗{\overset{˘}{T}}^{1}$ and $\text{M}\overset{˘}{D}(\text{M})⊗{\overset{˘}{S}}^{1}⊗{\overset{˘}{T}}^{1}$ representations. A spin-bond-order-wave (SBOW) state is characterized by unequal canonical ensemble averages of the bond-centered up- and down-spin densities, 〈*c*_**n**:*A*(*i*)+_^†^ *c*_**n**′:*A*′(*i*′)+_〉_HF_ ≠ 〈*c*_**n**:*A*(*i*)−_^†^ *c*_**n**′:*A*′(*i*′)−_〉_HF_, where 〈⋯〉_HF_ means taking the quantum average in a HF eigenstate. They are mathematically interesting but of no occurrence under any realistic parameterization [36]. The rest are spin-density-wave states, including both ferromagnetism (FM) and antiferromagnetism (AFM). The antiferromagnetic ordering patterns are also classified into three groups in the same manner as the charge-ordering ones: AFM on the Cu sublattice (Γ-Cu-AFM), AFM on the Mo sublattice [Γ-Mo-AFM, M-Mo-AFM(1), and M-Mo-AFM(2)], and AFM on both the Mo and Cu sublattices [M-MoCu-AFM(1) and M-MoCu-AFM(2)]. These magnetic states are likely to be stabilized by the strong on-site Coulomb repulsions *U*_Mo_ and *U*_Cu_. Since all the possible magnetic states except the FM state have no macroscopic magnetization, major states before and after photoirradiation are to be the PM and FM states, respectively.

## 3. Competing Ground States

**Figure 3:**
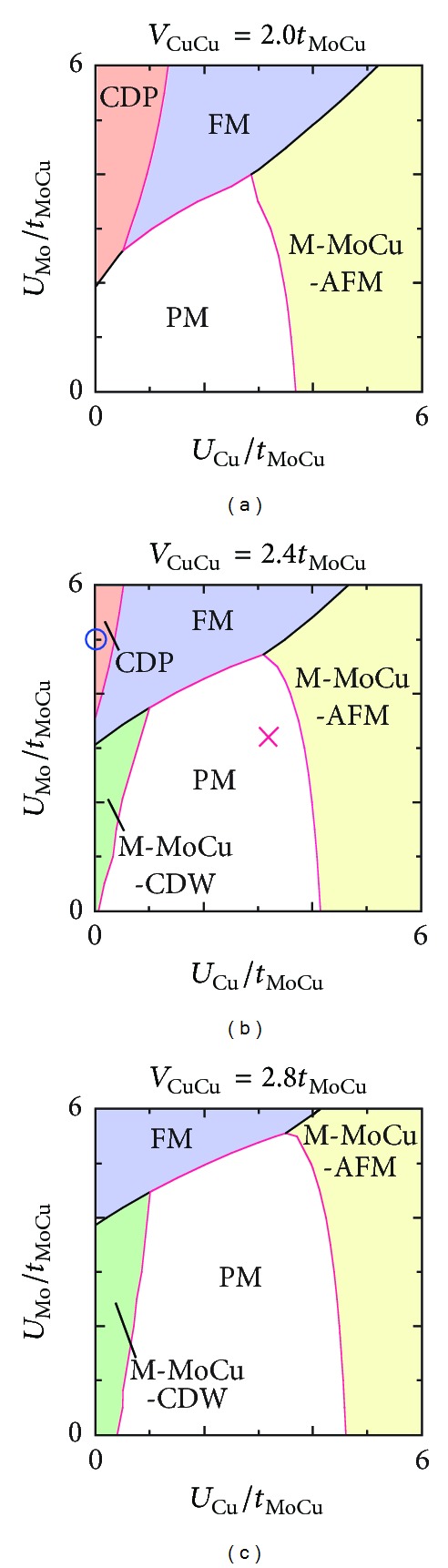
Ground-state phase diagrams as functions of the on-site Coulomb repulsions *U*_Cu_ and *U*_Mo_, where *t*_CuCu_ = 0.3*t*_MoCu_, *V*_MoCu_ = 0.8*t*_MoCu_, *ε*_Cu_ − *ε*_Mo_ = 1.0*t*_MoCu_, and *J*_MoCu_ = *J*_MoCu_′ = −*J*_CuCu_ = −*J*_CuCu_′ = 0.6*t*_MoCu_. M-MoCu-AFM and M-MoCu-CDW each include two isoenergetic phases of *A*_*g*_ and *B*_*g*_ symmetry, referred to as (1) and (2), respectively. Phase boundaries drawn in black and red denote transitions of the first and second order, respectively. We calculate optical properties of the PM state labeled ×. The CDP state labeled ∘ is discussed later in connection with photoexcitation of the PM state.

We have calculated the ground-state phase diagram at the sufficiently low temperature *k*_B_ *T* = 0.001*t*_MoCu_. In Figure 3, the magnetic states FM and M-MoCu-AFM are stabilized with *U*_Mo_ and *U*_Cu_ increasing. With increasing *V*_CuCu_, the nonmagnetic phases PM and M-MoCu-CDW grow up but the magnetic regions shrink. The exchange interactions *J*_MoCu_ and *J*_CuCu_ are most effective for the magnetic states. We learn from the identity ∑_*σ*,*τ*_ *c*_**n**:*A*(*i*)*σ*_^†^ *c*_**m**:*A*′(*i*′)*τ*_^†^ *c*_**n**:*A*(*i*)*τ*_ *c*_**m**:*A*′(*i*′)*σ*_ = −2**s**_**n**:*A*(*i*)_ · **s**_**m**:*A*′(*i*′)_ − *n*_**n**:*A*(*i*)_ *n*_**m**:*A*′(*i*′)_/2, where *s*_**n**:*A*(*i*)_^*λ*^ = ∑_*σ*,*σ*′_ *c*_**n**:*A*(*i*)*σ*_^†^ *c*_**n**:*A*(*i*)*σ*′_ *τ*_*σσ*′_^*λ*^/2. Considering that the analogue compound Cu_1.5_^II^[Mo^V^(CN)_8_] · 3H_2_O takes a ferromagnetic interaction between spins on Mo and Cu sites [26], we set positive *J*_MoCu_. On the other hand, the direct Cu-Cu exchange interaction *J*_CuCu_ is taken to be negative. The magnitude of the exchange interactions in the transfer-integral unit is comparable to that in typical cyano-bridged transition-metal complexes [37–39]. The pair-hopping correlation *J*_MoCu_′ set positive indirectly supports FM, destabilizing any other phase [27]. On the other hand, *J*_CuCu_′ set negative favors PM and M-MoCu-CDW because the pair hoppings between Cu sites are possible in them. PM and FM are sitting adjacently on the phase diagrams, and, therefore, a photoinduced phase transition between them is promising. With the experimental observations [26] in mind, we choose *U*_Cu_ = *U*_Mo_ = 3.2*t*_MoCu_ and *V*_CuCu_ = 2.4*t*_MoCu_ as standard parameters for Cu_2_^II^[Mo^IV^(CN)_8_], which is indicated by × in Figure 3. On this parameter point, we can well reproduce the valences of Mo^IV^ and Cu^II^ in the paramagnetic ground state. Another nonmagnetic and uniform state, referred to as CDP, can be stabilized in a *U*_Mo_ ≫ *U*_Cu_ region. In contrast with PM, CDP shows localization of electrons on Cu *d* orbitals. The PM and CDP states belong to the same irreducible representation but their electronic structures are quite different from each other. The details of CDP are discussed later.

**Figure 4:**
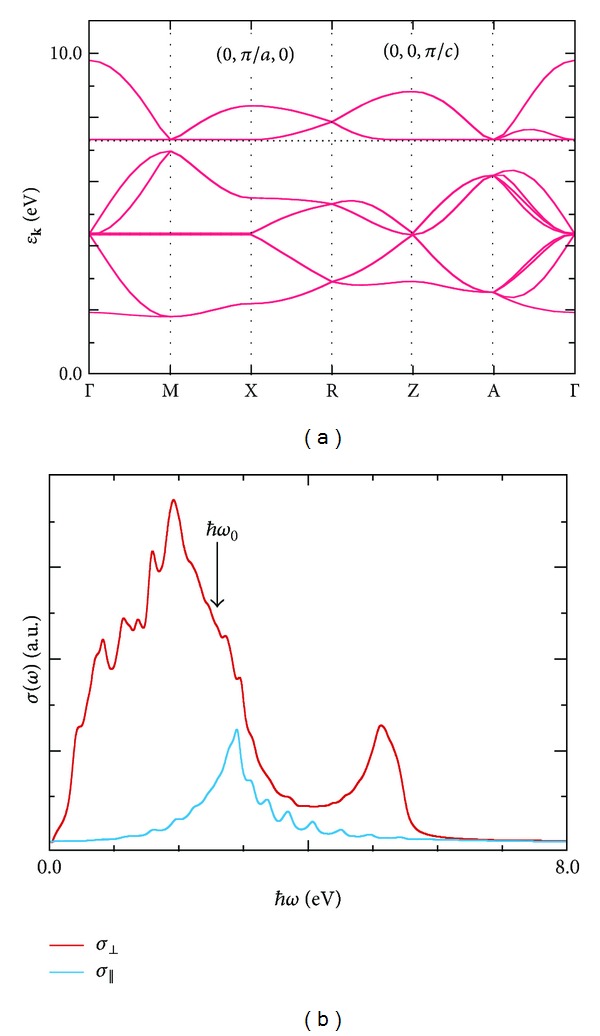
(a) An energy dispersion relation and (b) polarized optical conductivity spectra of the PM state. The Fermi energy *ε*_F_ is indicated by a dotted horizontal line. The optical conductivity spectra perpendicular and parallel to the *c* direction are given by *σ*_⊥_(*ω*) = *σ*_*a*_(*ω*) + *σ*_*b*_(*ω*) and *σ*_||_(*ω*) = *σ*_*c*_(*ω*), respectively. An arrow indicates a photon energy of pumping light, which is discussed in Section 4.

In Figure 4(a), we present a band structure of the PM state. Hereafter, the transfer integral *t*_MoCu_ is set equal to 0.7 eV, which is comparable to the intermetallic transfer integrals in typical Prussian-blue analogues [7, 40]. In the PM state, bands for the up- and down-spin electrons are degenerate and the Fermi level lies in the band gap of 0.35 eV. Thus it exhibits insulating behavior. In the PM state, the Cu *d* orbitals contribute to both the occupied and unoccupied bands, whereas the Mo *d* orbitals contribute only to the occupied bands. One can easily understand these differences by considering the valences of Cu and Mo ions in the PM state.

We further calculate the polarized optical conductivity defined as

(4) $$\begin{array}{c} \sigma_{\lambda }\left(\omega \right)=\sigma_{\lambda }^{0}\delta \left(\omega \right)+\sigma_{\lambda }^{\text{reg}}\left(\omega \right), \end{array}$$

where

(5) $$\begin{array}{c} \sigma_{\lambda }^{\text{reg}}\left(\omega \right)=\frac{\pi }{N\omega }\underset{i\neq 0}{\sum }{\left|\langle E_{i}\;|\;J_{\lambda }\;|\;E_{0}\rangle \right|}^{2}\delta \left(E_{i}-E_{0}-\hbar \omega \right) \end{array}$$

with the relevant current operator

(6) Jλ=i∑〈n,m,i,j〉∑σerCu(i);  Mo(j)λℏ×{[(−1)i+j+1tMoCu−VMoCupn:Cu(i);m:Mo(j)σ∗+JMoCupn:Cu(i);m:Mo(j)∗+JMoCu′pn:Cu(i);m:Mo(j)−σ]×cn:Cu(i)σ†cm:Mo(j)σ} +i∑〈n≠m〉 ∑i=14 ‍∑σerCu(i);  Cu(i+4)λℏ×{[tCuCu−VCuCupn:Cu(i);m:Cu(i+4)σ∗+JCuCupn:Cu(i);m:Cu(i+4)∗+JCuCu′pn:Cu(i);m:Cu(i+4)−σ]×cn:Cu(i)σ†cm:Cu(i+4)σ}+H.c.

Here, |*E*_*i*_〉 is an arbitrary wave function of energy *E*_*i*_ (*E*_0_ ≤ *E*_1_ ≤ *E*_2_ ≤ ⋯), *r*_*A*(*i*);*A*′(*i*′)_^*λ*^ indicates the *λ*( = *a*, *b*, *c*)-direction component of the vector **r**_*A*(*i*);*A*′(*i*′)_ which is the relative vector between nearest neighboring *A*(*i*) and *A*′(*i*′) sites, and *p*_**n**:*A*(*i*);**n**′:*A*′(*i*′)_^*σ*^ ≡ 〈*c*_**n**:*A*(*i*)*σ*_^†^ *c*_**n**′:*A*′(*i*′)*σ*_〉_HF_. Hereafter, we set |**c**| equal to |**a**|. The Drude spectral weight [41, 42] in (4) is given by

(7) $$\begin{array}{c} \sigma_{\lambda }^{0}=-\frac{\pi }{N}\left(\langle E_{0}\;|\;T_{\lambda }\;|\;E_{0}\rangle +2\underset{i\neq 0}{\sum }\frac{{\left|\langle E_{i}\;|\;J_{\lambda }\;|\;E_{0}\rangle \right|}^{2}}{E_{i}-E_{0}}\right) \end{array}$$

with the kinetic-energy operator

(8) Tλ=∑〈n,m,i,j〉 ∑σ[erCu(i);  Mo(j)λℏ]2×{[(−1)i+j+1tMoCu−VMoCupn:Cu(i);m:Mo(j)σ∗+JMoCupn:Cu(i);m:Mo(j)∗+JMoCu′pn:Cu(i);m:Mo(j)−σ]×cn:Cu(i)σ†cm:Mo(j)σ} +∑〈n≠m〉 ∑i=14 ‍∑σ[erCu(i);  Cu(i+4)λℏ]2×{[tCuCu−VCuCupn:Cu(i);m:Cu(i+4)σ∗+JCuCupn:Cu(i);m:Cu(i+4)∗+JCuCu′pn:Cu(i);m:Cu(i+4)−σ]×cn:Cu(i)σ†cm:Cu(i+4)σ}+H.c.

We define the ground state |*E*_0_〉 as

(9) $$\begin{array}{c} |E_{0}\rangle =\underset{\varepsilon_{k(\chi )\sigma }\leq \varepsilon_{\text{F}}}{\prod }c_{k(\chi )\sigma }^{†}|0\rangle , \end{array}$$

where |0〉 is the true electron vacuum, *ε*_F_ is the Fermi energy, and *c*_**k**(*χ*)*σ*_^†^ creates an electron of spin *σ* in the HF eigenstate with an eigenvalue *ε*_**k**(*χ*)*σ*_ (*χ* = 1,2,…, 12). Excited states can be calculated within the HF scheme, being generally defined as

(10) $$\begin{array}{c} |E_{i}\rangle =c_{k(\nu )\sigma }^{†}c_{k(\mu )\sigma }|E_{0}\rangle , \end{array}$$

where *ε*_**k**(*μ*)*σ*_ ≤ *ε*_F_ < *ε*_**k**(*ν*)*σ*_. Every excited state of the HF type is a single Slater determinant.

The thus-calculated absorption spectra are shown in Figure 4(b), where every spectral line is Lorentzian-broadened by a width of 0.1*t*_MoCu_. The PM state shows gap-full structures in the *σ*_⊥_ and *σ*_||_ spectra. A major absorption in *σ*_||_ is attributed to electron excitations from the highest-occupied molecular orbital (HOMO) to the lowest-unoccupied molecular orbital (LUMO) at point Γ, while a broad absorption band in *σ*_⊥_ is due to the HOMO-LUMO interband transitions at around points Γ, M, and A. *σ*_⊥_ has a larger oscillator strength than that of *σ*_||_ by the Cu-to-Cu electron transfers in the *a*-*b* plane.

## 4. Phototunable Magnetism

Here we investigate effects of photoirradiation of the PM ground state. The photoinduced magnetization is hardly detectable at zero magnetic field, while it rapidly increases just as an external field is applied [22]. With this in mind, we apply a moderate field to the system as follows:

(11) $$\begin{array}{c} H_{\text{ex}}=-g\mu_{\text{B}}H\underset{n}{\sum }\left[\overset{8}{\underset{i=1}{\sum }}s_{n:\text{Cu}(i)}^{c}+\overset{4}{\underset{j=1}{\sum }}s_{n:\text{Mo}(j)}^{c}\right], \end{array}$$

where *H* = 2.5 T. Now the spin degeneracy is lifted and higher-lying down-spin electrons may selectively be excited into conduction bands. We describe such photoexcitations by multiplying the hopping term *c*_**n**:*A*(*i*)−_^†^ *c*_**n**′:*A*′(*i*′)−_ by the Peierls phase factor exp⁡[−(i*e*/*ℏv*)**A**(*t*) · **r**_*A*(*i*);*A*′(*i*′)_] [43], where *e* and *v* are the elementary charge and the light velocity, respectively. Any spatial variation of the vector potential **A**(*t*) is negligible for visible lights. With the experimental observations [26] in mind, we set the pumping photon energy *ℏω*_0_ equal to 2.6 eV unless otherwise noted, which is indicated by the vertical arrow in Figure 4. Another key ingredient of the photomagnetization is a spin-mixing interaction. In an attempt at breaking the conservation law for the total magnetization, we introduce Dzyaloshinskii-Moriya (DM) interactions:

(12) HDM=∑n ∑l=14 ∑ρ=01 ∑σ,σ′=01(−1)ρ+σDl+4ρ(σ′σ) ·[sn+δ(l,ρ,σ,σ′):Mo(1+3σ′+(−1)σ′ρ)×sn:Cu(2l−σ)],

where **δ**(*l*, *ρ*, *σ*, *σ*′) = *σ*′*Re*[*f*(*l*)](**a**/*a*) + *σ*′*Im*⁡[*f*(*l*)](**b**/*a*) + *σρ*(**c**/*c*) with $f(l)=e^{\text{i}\pi /4}[1+e^{\text{i}\pi (1-l)/2}]/\sqrt{2}$. The DM vectors should be compatible with the crystalline structure as **D**_*i*_^(*σ*′*σ*)^ = *g*_*i*_ · **D**_1_^(*σ*′*σ*)^ with *g*_*i*_(∈**C**_4*h*_) = *C*_4*z*_^−1^, *C*_2*z*_, *C*_4*z*_, *σ*_*h*_, *IC*_4*z*_, *I*, *IC*_4*z*_^−1^ for *i* = 2 to 8, respectively. We lay them down in the *ab* plane with **D**_1_^(*σ*′*σ*)^ = (*D*, 0,0). In order to visualize photoinduced electronic excitations and the following magnetic relaxation, we solve the time-dependent Schrödinger equation:

(13) $$\begin{array}{c} \text{i}\hbar \dot{\Psi }\left(t\right)=H_{\text{HF}}\left(t\right)\Psi \left(t\right), \end{array}$$

where the instantaneous Hamiltonian *H*_HF_(*t*) (*t* ≥ 0) consists of *H*, *H*_ex_, and *H*_DM_ in the Hartree-Fock approximation [44, 45] with the Peierls phase factor switched on, while the path-integrated wave-function array Ψ(*t*) is a square matrix of degree 24*N* and we define Ψ(0) as the complete set of wave functions for the “static” Hamiltonian, {Ψ_**k**(*ν*)_(0); **k**(*ν*) = 1,…, 24*N*}, which are specified with momentum **k** and band label *ν*. Discretizing the time variable as *t*_*m*_ = *m*Δ*t*  (*m* = 0,1, 2,…) with Δ*t* = 0.005*ℏ*/*t*_MoCu_, we integrate (13) step by step:

(14) $$\begin{array}{c} \Psi \left(t_{m+1}\right)=\exp\left[-\frac{\text{i}H_{\text{HF}}\left(t_{m}\right)}{\hbar }\Delta t\right]\Psi \left(t_{m}\right). \end{array}$$

Once we start the time integration (*m* > 0), Ψ(*t*_*m*_) deviates from the eigenvectors of *H*_HF_(*t*_*m*_) in general. Pump laser pulses are described by the vector potential,

(15) $$\begin{array}{c} A\left(t\right)=A_{0}\overset{3}{\underset{i=0}{\sum }}e^{-\gamma^{2}{\left(t-t_{i}\right)}^{2}}\cos\omega_{i}t, \end{array}$$

where centers and photon energies of the first~fourth (*i* = 0 ~  3) pulses are given by *t*_*i*_ = 0.38 + 0.75*i* ps and *ℏω*_*i*_ = (1 − 0.25*i*)*ℏω*_0_, respectively, and a width and an amplitude of each pulse are 2*γ* ^−1^ = 0.25 ps and |**A**_0_ | = *A*_0_ = 2.0(*ℏv*/*ea*), respectively. We irradiate the PM state with *a*-axis-polarized [**A**_0_ = (*A*_0_, 0,0)] and *c*-axis-polarized [**A**_0_ = (0,0, *A*_0_)] photons.

**Figure 5:**
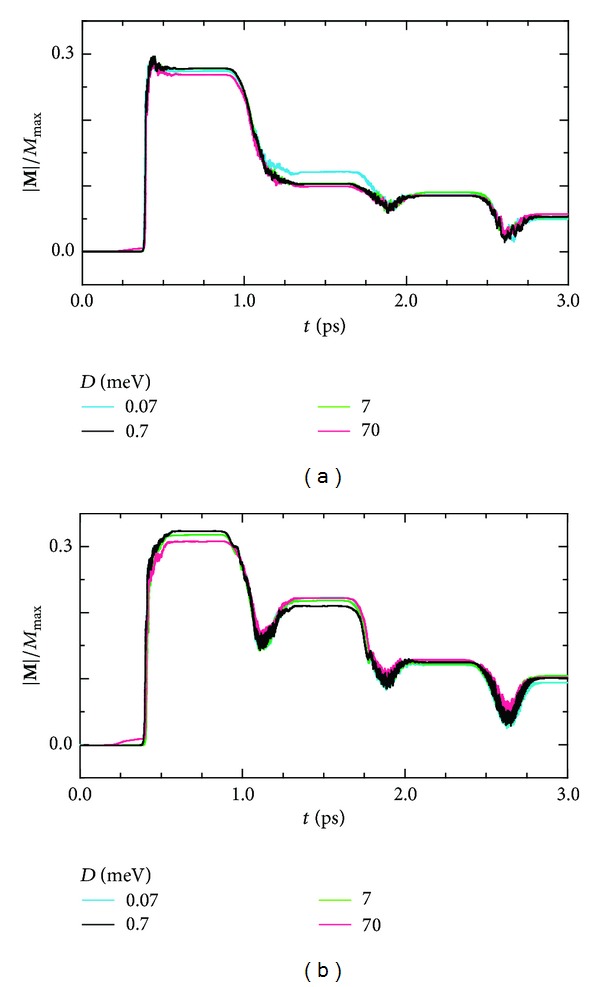
Manipulation of the magnetization by applying successive pump laser pulses in the form of (15) with (a) **A**_0_||**a** and (b) **A**_0_||**c** in the presence of the DM interaction (*D* = 0.07,0.7,7, 70 meV), where *M*_max⁡_ is the saturated magnetization.

In Figure 5, we study DM interaction effects on the photomagnetism, before entering into detailed discussions of the dynamics. The *λ*( = *a*, *b*, *c*)-direction component of the magnetization vector **M** is defined by

(16) $$\begin{array}{c} M^{\lambda }=\frac{1}{N}\underset{n}{\sum }\langle \Phi \left(t\right)|\left|\left(\overset{4}{\underset{i=1}{\sum }}s_{n:\text{Mo}\left(i\right)}^{\lambda }+\overset{8}{\underset{i=1}{\sum }}s_{n:\text{Cu}\left(i\right)}^{\lambda }\right)\right||\Phi \left(t\right)\rangle \end{array}$$

with |Φ(*t*)〉 = ∏_**k**(*ν*)=1_^16*N*^ ⊗ |Ψ_**k**(*ν*)_(*t*)〉, as a function of time. Both the *a*- and *c*-axis-polarized photons indeed induce magnetization and demagnetization of the system. The macroscopic magnetizations are induced by the first pulses and they are erased successively by the second, third, and fourth pulses. The induced magnetizations by the *c*-axis-polarized photons are slightly larger than those by the *a*-axis-polarized ones. Even though *D* varies over three orders of magnitude (0.07–70 meV), the induced magnetizations take almost the same value and the following demagnetization processes hardly vary. Depending on the value of *D*, the global magnetization occurs at different points in time, but these differences are at most 9 fs and 14 fs in the cases of **A**_0_||**a** and **A**_0_||**c**, respectively. Of course, in the absence of the DM interaction, photoirradiation never induces a net magnetization. Thus, the DM interaction plays an important role to bring the magnetic relaxation but does not so strongly affect the performances of the photomagnets. Hereafter, we set the magnitude of the DM vector equal to 0.7 meV, which is comparable to that in typical transition-metal oxides [46, 47].

**Figure 6:**
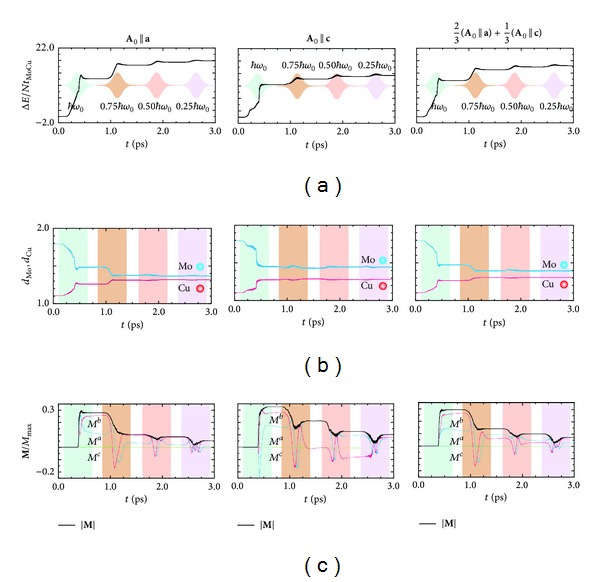
Manipulation of the magnetization by applying successive pump laser pulses in the form of (15) with **A**_0_||**a** (the left panels) and **A**_0_||**c** (the middle panels). (a) An absorbed photon energy and an envelope of the applied vector potential. (b) Averaged electron densities on Mo and Cu sites. (c) A magnetization. The two-to-one mixtures of the results in the cases of **A**_0_||**a** and **A**_0_||**c** (the right panels) are also presented in an attempt to reproduce powder-sample observations [26].

In Figure 6, we present details of the photoinduced dynamics, where the increase of the electronic energy,

(17) $$\begin{array}{c} \Delta E=\langle \Phi \left(t\right)|H_{\text{HF}}\left(t\right)|\Phi \left(t\right)\rangle -\langle \Phi \left(0\right)|H_{\text{HF}}|\Phi \left(0\right)\rangle , \end{array}$$

the averaged electron densities on Mo and Cu sites,

(18) dMo=14N∑i=14 ∑n〈Φ(t)|nn:Mo(i)|Φ(t)〉,dCu=18N∑i=18 ∑n〈Φ(t)|nn:Cu(i)|Φ(t)〉,

and the magnetization, **M**, are monitored. We first focus on the dynamics induced by the *a*-axis-polarized photons. The irradiation with *ℏω*_0_ photons first induces electron transfers from Mo to Cu sites. The electronic energy nonmonotonically increases with a time passage through instantaneous stagnation in the photoabsorption, which is seen at around the center of the pulse. Δ*E* upon the first photoirradiation reads as a double-stepped function of time. The occurrence of the significant magnetization looks simultaneous with the second step of Δ*E*(*t*). The thus-induced magnetization is decreased by the second irradiation with 0.75*ℏω*_0_ photons. Even in this demagnetization process, electrons further transfer from Mo to Cu sites. This means that the demagnetization process cannot be interpreted as a simple reverse process of the magnetization. The magnetization gets smaller and smaller by the second, third, and fourth pulses; that is, the photoinduced ferromagnetic state comes back to a nonmagnetic state finally. Next, we discuss the case of **A**_0_||**c**. We obtain qualitatively the same results with **A**_0_||**a**, upon the first photoirradiation. However, the absorbed energy is slightly smaller than that in the case of **A**_0_||**a**. Given the larger magnetization than that induced by the *a*-axis-polarized photons, the *c*-axis-polarized ones are more effective for photomagnetization than the *a*-axis-polarized ones. Electron transfers upon the subsequent photoirradiation are insignificant in comparison with those in the case of **A**_0_||**a**, where the electrons transiently come back to Mo sites only by the third photoirradiation. However, step-by-step demagnetization, observed in the case of **A**_0_||**a** as well, is clearly seen. Thus, the dynamics induced by the *a*- and *c*-axis-polarized photons are similar to each other. Even if we consider superposition of the two excited states which are induced by photons polarized in the *a* and *c* directions (see the right panels of Figure 6), the above magnetic features are retained. The tunable photomagnetism we revealed here agrees with the polycrystalline-sample measurements [26]. We further present *M* ^*a*^, *M* ^*b*^, and *M* ^*c*^ corresponding to the *a*-, *b*-, and *c*-direction components of **M**, respectively, in Figure 6(c). In both the cases of **A**_0_||**a** and **A**_0_||**c**, photoirradiation does not induce *M* ^*c*^. While rotating in the *a*-*b* plane, the induced magnetizations disappear. Here, we emphasize that the thus-demagnetized states by the photoirradiation differ from the initial stationary PM state in the valence arrangement. The electron densities on both Cu and Mo sites come close to the average value 16/12 = 1.33.

**Figure 7:**
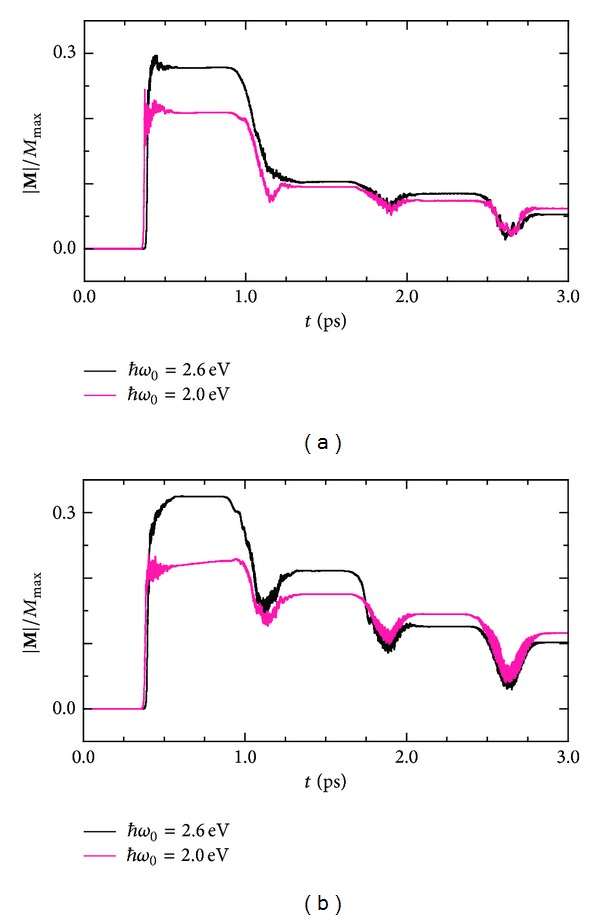
Manipulation of the magnetization by applying successive pump laser pulses in the form of (15) with (a) **A**_0_||**a** and (b) **A**_0_||**c**, where the photon energy *ℏω*_0_ is set equal either to 2.0 eV or to 2.6 eV.

Photon-energy dependences of the magnetization and demagnetization dynamics are shown in Figure 7. In the case of **A**_0_||**a**, the induced magnetization value decreases with decreasing irradiated photon energy. However, the magnetization values in the final steady states at 3.0 ps seem to be unaffected by the photon energy *ℏω*_0_. The same tendency is seen in the dynamics induced by the *c*-axis-polarized photons. Here, we recall the ground-state optical conductivity of PM shown in Figure 4(b). In the *σ*_||_ spectrum, an absorption at 2.0 eV is smaller than that at 2.6 eV, whereas, in the *σ*_⊥_ spectrum, the former is larger than the latter. Despite such differences between *σ*_⊥_ and *σ*_||_, the magnetic responses to the *a*- and *c*-axis-polarized photons resemble each other in the photon-energy dependence. The photoinduced ferromagnetism is strongly affected by the photon energy itself rather than the intensity of the corresponding photon absorption. We will thus be able to control a sensitivity of the photomagnetism by tuning a wavelength of the irradiation light.

In order to see changes in the electronic structures in more detail, we calculate one-particle spectral functions [48]:

(19) $$\begin{array}{c} D\left(t;k,\omega \right)=E\left(t;k,\omega \right)+H\left(t;k,\omega \right) \end{array}$$

with the electron part

(20) $$\begin{array}{c} E\left(t;k,\omega \right)=\overset{24N}{\underset{k\left(\nu \right)=1}{\sum }}\delta \left(\hbar \omega -\varepsilon_{k(\nu )}\right)\langle \Phi \left(t\right)|{\tilde{c}}_{k\left(\nu \right)}^{†}{\tilde{c}}_{k(\nu )}|\Phi \left(t\right)\rangle ,\;\; \end{array}$$

and the hole part

(21) $$\begin{array}{c} H\left(t;k,\omega \right)=\overset{24N}{\underset{k\left(\nu \right)=1}{\sum }}\delta \left(\hbar \omega -\varepsilon_{k(\nu )}\right)\langle \Phi \left(t\right)|{\tilde{c}}_{k(\nu )}{\tilde{c}}_{k\left(\nu \right)}^{†}|\Phi \left(t\right)\rangle . \end{array}$$

**Figure 8:**
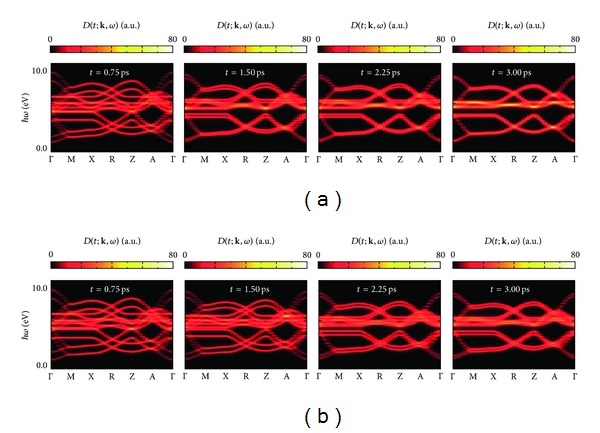
Snapshots of the one-particle spectral function *D*(*t*; **k**, *ω*) every time we apply a pump laser pulse in the form of (15) in the cases of **A**_0_||**a** (the upper panels) and **A**_0_||**c** (the lower panels).

Here, ${\tilde{c}}_{k(\nu )}^{†}$ creates an electron in the eigenstate of energy *ε*_**k**(*ν*)_ for the instantaneous Hamiltonian *H*_HF_(*t*) and the delta function is Lorentzian-broadened with a width of 0.1*t*_MoCu_.  Figure 8 visualizes *D*(*t*; **k**, *ω*) corresponding to “momentum-resolved” density of states in the cases of irradiating the PM state with the pulsed laser lights polarized parallel to the crystalline axes *a* and *c*, which correspond to the time variations in Figure 6. They demonstrate time evolutions of the band structure of the PM state shown in Figure 4(a). As is shown in Figure 8, band structure changes in the cases of **A**_0_||**a** and **A**_0_||**c** resemble each other. In the photoinduced FM states at *t* = 0.75 ps, the degeneracy between up and down spins in the initial stationary PM state is so lifted as to induce a net magnetization. The band splitting caused by the *c*-axis-polarized photons is more noticeable than that by the *a*-axis-polarized ones. That is why the larger magnetization is induced by the *c*-axis-polarized photons. In the subsequent demagnetization processes, the up- and down-spin bands intend to converge, where we find stimulated photon emission as well as photon absorption, which is never seen in the magnetization processes. A significant decrease of the band splitting is induced by the second pulse irradiation with *a*-axis-polarized photons, which corresponds to the remarkable demagnetization shown in Figure 6(c). The induced magnetizations decrease with decreasing band splitting, by the third and fourth photoirradiation. The thus-demagnetized final steady states at *t* = 3.00 ps show an entirely different band structure from that of the initial stationary PM state.

## 5. Summary and Discussion

We have revealed phototunable magnetism in the cyano-bridged copper-molybdenum bimetallic assembly Cu_2_[Mo(CN)_8_], where the coordination geometry of every molybdenum complex ion is square antiprism. Indeed the magnetization is both induced and erased optically, but the present reversible magnetism does not necessarily mean a round trip of electrons. Divalent copper ions are reduced into monovalent ones during magnetization, while reoxidation of these photoexcited copper ions is of rare occurrence during demagnetization. Electrons of Cu character and those of Mo character are both fully itinerant in any of the photoinduced steady states and look different from rather atomic ones in the initial equilibrium paramagnetic state. In this context we may be reminded of the thus-called hidden states [49].

**Figure 9:**
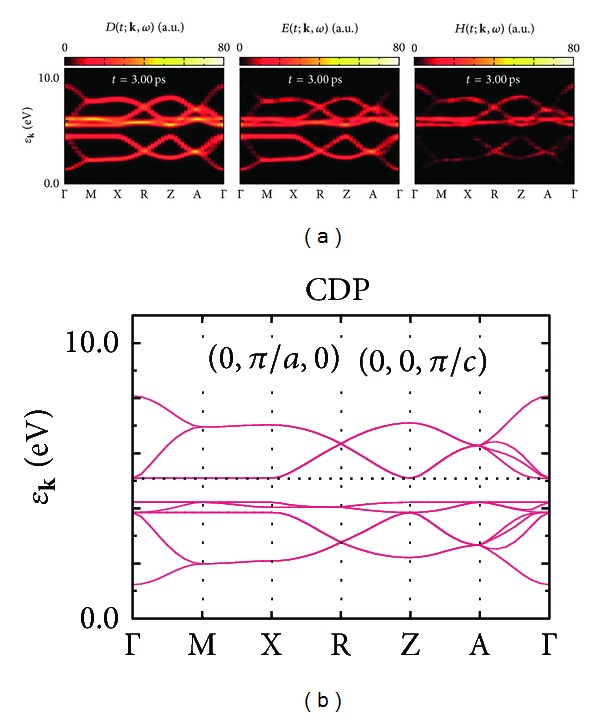
(a) The one-particle spectral functions *D*(*t*; **k**, *ω*), *E*(*t*; **k**, *ω*), and *H*(*t*; **k**, *ω*) at *t* = 3.00 ps in the case of **A**_0_||**a** in Figure 6. (b) A band structure of the CDP state labeled ∘ in Figure 3. The Fermi energy *ε*_F_ is indicated by a dotted horizontal line.

Figure 9(a) visualizes the density of states *D*(*t*; **k**, *ω*), the electron-distribution function *E*(*t*; **k**, *ω*), and the hole-distribution function *H*(*t*; **k**, *ω*) of the demagnetized final steady state at *t* = 3.00 ps. This band structure looks very similar to that of the equilibrium CDP state shown in Figure 9(b). The CDP state exhibits insulating behavior due to the energy gap of 0.88 eV, where the Cu and Mo *d* orbitals contribute to the occupied and unoccupied bands, respectively, because all the electrons are localized on Cu sites. On the other hand, the final steady state has no well-defined electron and hole bands anymore due to the photoirradiation, which are clearly seen in *E*(*t*; **k**, *ω*) and *H*(*t*; **k**, *ω*), and therefore this state is metallic. Electron transfers from Cu to Mo sites in the equilibrium CDP state can lead to situation just like Figure 9(a). Thus the final steady state may read as a CDP state which has the different electron distribution from that in the equilibrium situation. The photoconverted FM state from the PM state is further optically converted to such a “nonequilibrium” CDP state. In the ground-state phase diagram, the CDP phase as well as the PM phase is indeed located adjacent to the FM phase (*cf*.  Figure 3(b)). Such a relationship among three phases may be a key ingredient of the tunable photomagnetism. In order to improve a performance of the photomagnets, it is also necessary to clarify thermal and chemical-doping effects on the phase competition.

Modern experiments enable us to directly observe femtosecond electron transfer dynamics [50–54]. For example, time-resolved optical measurements can detect the metallic conductivity of the photoexcited states and time- and angle-resolved photoemission measurements can directly observe the changes in the band structures shown in Figure 8. We hope the present calculations will stimulate such ultrafast pump-probe experiments on the cyano-bridged copper-molybdenum bimetallic assemblies.

## Figures and Tables

**Table 1:** Symmetry properties of the irreducible representations, $\Gamma \overset{ˇ}{D}(\Gamma )⊗{\overset{ˇ}{S}}^{0}⊗{\overset{ˇ}{T}}^{0}$, M$\overset{ˇ}{D}(\text{M})⊗{\overset{ˇ}{S}}^{0}⊗{\overset{ˇ}{T}}^{0}$, $\Gamma \overset{ˇ}{D}(\Gamma )⊗{\overset{ˇ}{S}}^{1}⊗{\overset{ˇ}{T}}^{1}$, and M$\overset{ˇ}{D}(M)⊗{\overset{ˇ}{S}}^{1}⊗{\overset{ˇ}{T}}^{1}$, available in the Hamiltonian (1) subject to axial isotropy subgroup.

Irreducible representation	Axial isotropy subgroup	Physical character
$\Gamma A_{g}⊗{\overset{˘}{S}}^{0}⊗{\overset{˘}{T}}^{0}$	**C**_4*h*_ **L**_1_ST	PM, CDP
$\Gamma B_{g}⊗{\overset{˘}{S}}^{0}⊗{\overset{˘}{T}}^{0}$	**C**_2*h*_ **L**_1_ST	Γ-Cu-CDW
$\Gamma A_{u}⊗{\overset{˘}{S}}^{0}⊗{\overset{˘}{T}}^{0}$	**C**_4_ **L**_1_ST	Γ-Mo-CDW
$\Gamma B_{u}⊗{\overset{˘}{S}}^{0}⊗{\overset{˘}{T}}^{0}$	**S**_4_ **L**_1_ST	BOW

M$A_{g}⊗{\overset{˘}{S}}^{0}⊗{\overset{˘}{T}}^{0}$	**C**_4*h*_ **L**_2_ST	M-MoCu-CDW(1)
M$B_{g}⊗{\overset{˘}{S}}^{0}⊗{\overset{˘}{T}}^{0}$	(*E* + *C*_4*z*_^+^ **a**)**C**_2*h*_ **L**_2_ST	M-MoCu-CDW(2)
M$A_{u}⊗{\overset{˘}{S}}^{0}⊗{\overset{˘}{T}}^{0}$	(*E* + *I * **a**)**C**_4_ **L**_2_ST	M-Mo-CDW(1)
M$B_{u}⊗{\overset{˘}{S}}^{0}⊗{\overset{˘}{T}}^{0}$	(*E* + *C*_4*z*_^+^ **a**)**S**_4_ **L**_2_ST	M-Mo-CDW(2)

$\Gamma A_{g}⊗{\overset{˘}{S}}^{1}⊗{\overset{˘}{T}}^{1}$	**C**_4*h*_ **L**_1_A(*e*_*z*_)M(*e*_*y*_)	FM
$\Gamma B_{g}⊗{\overset{˘}{S}}^{1}⊗{\overset{˘}{T}}^{1}$	(*E* + *C*_4*z*_^+^ *u*_2*x*_)**C**_2*h*_ **L**_1_A(*e*_*z*_)M(*e*_*y*_)	Γ-Cu-AFM
$\Gamma A_{u}⊗{\overset{˘}{S}}^{1}⊗{\overset{˘}{T}}^{1}$	(*E* + *Iu*_2*x*_)**C**_4_ **L**_1_A(*e*_*z*_)M(*e*_*y*_)	Γ-Mo-AFM
$\Gamma B_{u}⊗{\overset{˘}{S}}^{1}⊗{\overset{˘}{T}}^{1}$	(*E* + *C*_4*z*_^+^ *u*_2*x*_)**S**_4_ **L**_1_A(*e*_*z*_)M(*e*_*y*_)	SBOW

M$A_{g}⊗{\overset{˘}{S}}^{1}⊗{\overset{˘}{T}}^{1}$	(*E* + *u*_2*x*_ **a**)**C**_4*h*_ **L**_2_A(*e*_*z*_)M(*e*_*y*_)	M-MoCu-AFM(1)
M$B_{g}⊗{\overset{˘}{S}}^{1}⊗{\overset{˘}{T}}^{1}$	(*E* + *u*_2*x*_ **a**)(*E* + *C*_4*z*_^+^ *u*_2*x*_)**C**_2*h*_ **L**_2_A(*e*_*z*_)M(*e*_*y*_)	M-MoCu-AFM(2)
M$A_{u}⊗{\overset{˘}{S}}^{1}⊗{\overset{˘}{T}}^{1}$	(*E* + *u*_2*x*_ **a**)(*E* + *Iu*_2*x*_)**C**_4_ **L**_2_A(*e*_*z*_)M(*e*_*y*_)	M-Mo-AFM(1)
M$B_{u}⊗{\overset{˘}{S}}^{1}⊗{\overset{˘}{T}}^{1}$	(*E* + *u*_2*x*_ **a**)(*E* + *C*_4*z*_^+^ *u*_2*x*_)**S**_4_ **L**_2_A(*e*_*z*_)M(*e*_*y*_)	M-Mo-AFM(2)

Consider **C**_4*h*_ = {*E*, *C*_4*z*_, *C*_2*z*_, *C*_4*z*_^−1^, *I*, *IC*_4*z*_, *σ*_*h*_, *IC*_4*z*_^−1^},  **C**_2*h*_ = {*E*, *C*_2*z*_, *I*, *σ*_*h*_},

**C**_4_ = {*E*, *C*_4*z*_, *C*_2*z*_, *C*_4*z*_^−1^},  **S**_4_ = {*E*, *C*_2*z*_, *IC*_4*z*_, *IC*_4*z*_^−1^},

**L**_1_ = {*l*_*a*_ **a** + *l*_*b*_ **b** + *l*_*c*_ **c**; *l*_*a*_, *l*_*b*_, *l*_*c*_ ∈ *Z*},  **L**_2_ = {(*l*_+_ + *l*_−_)**a** + (*l*_+_ − *l*_−_)**b** +  *l*_*c*_ **c**; *l*_+_, *l*_−_, *l*_*c*_ ∈ *Z*},

A(*e*_*z*_) = {*u*(*e*_*z*_, *θ*) | 0 ≤ *θ* ≤ 4*π*}, M(*e*_*λ*_) = {*E*, *tu*_2*λ*_},  *u*_2*λ*_ = *u*(*e*_*λ*_, *π*)∶*λ* = *x*, *y*, *z*.
